# THE RELATIONSHIP BETWEEN AGING PERCEPTIONS AND COGNITION AMONG OLDER ADULTS

**DOI:** 10.1093/geroni/igae098.3750

**Published:** 2024-12-31

**Authors:** Ava Mcvey, Christine Phillips, Lesley Ross

**Affiliations:** Clemson University, Clemson, South Carolina, United States; Clemson University Institute for Engaged Aging, Seneca, South Carolina, United States; Clemson University, Clemson, South Carolina, United States

## Abstract

Maintaining cognition is critical to overall health and independence. While behavioral predictors (e.g., cognitive engagement, social engagement, physical activity) of cognition well-established in research, the relationship of one’s perceptions of aging and cognition have been less thoroughly investigated. The current study cross-sectionally explored the relationships between self-perceptions of aging (SPA; including awareness of age-related change and expectations regarding aging) and cognition, including performance-based measures of executive function (Matrix Reasoning, Letter/Number Switching, Digit Symbol, Trails A & B, Visual Paired Associates) and self-reported cognition (Cognitive Self Report Questionnaire). Participants (n=334) included community dwelling older adults from the: Elucidating the Necessary Active Components of Training (ENACT) and Everyday Function Intervention Trials (E-FIT) studies. A series of four multivariate linear regressions were conducted, one for each facet of SPA and both executive function and self-reported cognition. Results found that neither awareness of age-related change nor expectations regarding aging predicted performance-based executive functioning. However, both awareness of age-related change (β =.65, p <.001, 95% CI [.29,1.03]) and expectations regarding aging (β =.17, p <.001, 95% CI [.11,.23]) predicted self-reported cognition. The current study addresses a gap in understanding how different facets of SPA may associate with different aspects of cognition (executive functioning and self-reported cognition). Results suggest that SPA do not correlate with objective executive functioning performance in this sample. However, one’s perception of the aging process may give insight into how they perceive their own current cognitive abilities, which can be an important indicator of well-being.

